# Strategies to Measure and Improve Emergency Department Performance: A Review

**DOI:** 10.7759/cureus.52879

**Published:** 2024-01-24

**Authors:** Reham Mostafa, Khaled El-Atawi

**Affiliations:** 1 Department of Emergency Medicine, Al Zahra Hospital Dubai (AZHD), Dubai, ARE; 2 Pediatrics/ Neonatal Intensive Care Unit, Latifa Women and Children Hospital, Dubai, ARE

**Keywords:** resources, length of stay, satisfaction, strategy, performance, emergency department

## Abstract

Emergency Departments (EDs) globally face escalating challenges such as overcrowding, resource limitations, and increased patient demand. This study aims to identify and analyze strategies to enhance the structural performance of EDs, with a focus on reducing overcrowding, optimizing resource allocation, and improving patient outcomes. Through a comprehensive review of the literature and observational studies, the research highlights the effectiveness of various approaches, including triage optimization, dynamic staffing, technological integration, and strategic resource management. Key findings indicate that tailored strategies, such as implementing advanced triage protocols and leveraging telemedicine, can significantly reduce wait times and enhance patient throughput. Furthermore, evidence suggests that dynamic staffing models and the integration of cutting-edge diagnostic tools contribute to operational efficiency and improved quality of care. These strategies, when combined, offer a multifaceted solution to the complex challenges faced by EDs, promising better patient care and satisfaction. The study underscores the need for a comprehensive approach, incorporating both organizational and technological innovations, to address the evolving needs of emergency healthcare.

## Introduction and background

Emergency Departments (EDs) are critical components of healthcare systems worldwide, functioning as the primary point of care for acute and urgent medical needs [1]. However, the increasing patient load and complex healthcare demands have put a significant strain on ED infrastructures, leading to challenges like overcrowding, prolonged wait times, and resource limitations [2]. High occupancy levels in EDs often correlate with prolonged waiting times, adversely impacting patient care quality and satisfaction [3].

Research has emphasized the importance of managing ED occupancy as a pivotal aspect of healthcare quality [4,5]. Furthermore, studies have shown that variability in admission rates reflects the diversity in patient demographics, hospital capabilities, and healthcare policies, thereby impacting ED performance [6]. The effective allocation and management of resources, including medical equipment and staffing, are crucial for reducing wait times and improving patient outcomes [7].

This study aimed to systematically explore various strategies to enhance ED performance, focusing on key areas such as reducing ED occupancy and crowding, optimizing admission rates, and improving resource management. By employing evidence-based approaches and integrating technological advancements, the study seeks to offer practical solutions for EDs to manage patient flow efficiently, ensure swift and accurate treatment, and ultimately enhance patient satisfaction and care quality. The strategies discussed in this study are grounded in extensive research and aim to address the multifaceted challenges faced by EDs in a rapidly evolving healthcare landscape.

## Review

Performance indicators of EDs

ED Structure

ED occupancy/crowding: ED occupancy or crowding is a crucial structural indicator reflecting the capacity of an ED to manage patient volume effectively. It is typically quantified by the number of patients in the waiting room, the percentage of ED beds filled, and the number of admitted patients awaiting inpatient beds [8]​​. Research suggests that crowding occurs when hospital occupancy exceeds 85%-90%, making it a critical factor to monitor [9]. High occupancy levels often correlate with prolonged waiting times and can significantly impact the quality of patient care. Crowding in the ED not only affects the timeliness and effectiveness of care but also patient safety and satisfaction [10]​​. It's a barometer for operational efficiency, highlighting the need for optimal resource allocation and efficient patient flow management within the ED. Ideally, occupancy rates should be moderate, allowing for efficient patient flow and reduced waiting times. However, the main challenge is unpredictably fluctuating patient volumes, leading to overcrowding and straining resources [5] (Table 1).

**Table 1:** Strategies to Improve ED Performance ED: Emergency Department

Domain	Key Strategies
ED Structure	
ED Occupancy/Crowding	Implement fast-track systems for minor ailments, advanced triage protocols, enhance coordination with other hospital departments, optimize staffing based on patient influx trends, and involve multidisciplinary teams.
Admission Rate	Use advanced diagnostic tools, integrate EHRs and telemedicine, enhance communication with inpatient units, and implement multidisciplinary teams for comprehensive assessment.
Resources	Flexible staffing models, use of lean management principles, adoption of advanced medical equipment and IT systems, telemedicine integration, and continuous staff training.
ED Process	
Time to Diagnosis, Treatment, Pain Management	Rapid assessment and triage protocols, point-of-care testing, decision support systems in EHRs, standardized treatment protocols, early pain assessment and treatment.
ED Length of Stay/Wait Time	Efficient triage systems, fast-track lanes for minor cases, optimizing staff efficiency, proactive pain management, rapid diagnostic and treatment procedures.
Correct Diagnosis and Appropriate Treatment	Advanced diagnostic equipment, continuous medical education, multidisciplinary case reviews, evidence-based treatment protocols, and specialist consultations.
Physician Workload	Align staffing with patient influx, use telemedicine, predictive analytics for resource planning, task delegation, wellness programs, and feedback-driven improvements.
Standard of Care Treatment	Adherence to clinical guidelines, benchmarking against best practices, regular training in current standards, and use of evidence-based protocols.
Quality of Care Measure	Regular monitoring and auditing, patient feedback surveys, staff training in quality improvement, and implementation of best practice guidelines.
ED Outcomes	
ED Returns	Robust discharge planning, clear follow-up instructions, scheduled follow-up calls or telehealth appointments, and comprehensive EHR system for tracking.
Left Without Being Seen (LWBS)	Rapid triage system, fast-track lanes, efficient staff allocation, enhanced communication about wait times, and comfortable waiting areas.
Mortality	Advanced resuscitation techniques, quick access to critical care resources, regular training, coordination with intensive care units, and continuous learning culture.
ED Satisfaction	
Patient Satisfaction	Reduce wait times, enhance communication skills, provide clear information, comfortable waiting areas, and patient-centered care approach.
Peer-Assessed Physician Performance	Regular peer reviews, feedback mechanisms, continuing medical education, and performance benchmarking.
Provider Communication	Effective internal communication channels, training in communication skills, regular team meetings, and feedback loops.
Rate of Complaints	Effective complaint management systems, patient feedback analysis, and continuous service improvement based on feedback.
Provider Satisfaction	Workload management, professional development opportunities, efficient administrative processes, and supportive work environment.
Staff Safety	Safety protocols and training, ergonomic workplace design, access to protective equipment, and mental health support.

A study highlighted the correlation between the four-hour standard, a benchmark for patient wait times and ED crowding, suggesting that this metric is a meaningful quality indicator in the context of emergency medicine [11]. Furthermore, a systematic review of 90 studies emphasized the variability in measuring ED crowding, underscoring the complexity of accurately assessing and managing this issue [12]. Additionally, research has linked high ED occupancy rates with reduced patient satisfaction, indicating a direct impact on patient perceptions of care quality [13]. In-depth studies have delved into the dynamics and consequences of ED occupancy and crowding. A pivotal study by Al-Qahtani in 2021 utilized ED occupancy rates to categorize crowding into distinct statuses such as 'crowding' and 'overcrowding'. This approach offers a nuanced perspective on how varying degrees of crowding impact patient experiences and outcomes. The investigation revealed that longer patient stays in the ED, often a result of high occupancy rates, are linked to a range of negative outcomes. These include extended waiting times, compromised patient care, and overall decreased efficiency of ED operations [4]. These studies collectively underline the importance of managing ED occupancy as a key component of healthcare quality. Addressing ED crowding is crucial not only for improving patient outcomes and satisfaction but also for enhancing the overall efficiency and effectiveness of emergency medical services.

Admission rate: The admission rate is another vital structural indicator, representing the proportion of ED visitors who require inpatient care. This rate is a measure of the severity and complexity of cases an ED handles. A higher admission rate may indicate a greater burden of serious conditions and the need for more robust inpatient resources and coordination [14]​​. It also impacts the flow within the ED, as admitted patients often require longer stays and more intensive care, potentially leading to increased crowding and resource strain [14]. A study by Venkatesh et al. revealed a broad range of admission rates across U.S. hospitals, with a median rate of 17.6%. This varied from as low as 7.8% to as high as 33% across different hospitals, highlighting the diversity in patient demographics, hospital capabilities, and healthcare policies that influence this rate [6]. The variation in admission rates can be a reflection of several factors, including the severity of illnesses treated in the ED, the availability of outpatient care and community resources, and the efficiency of the ED in triaging and managing patients. Lin et al. focused on trends in ED visits and admission rates among U.S. acute care hospitals. Their findings showed a significant change in these metrics over the study period. They observed that while there was an increase in the number of ED visits, the admission rates from the ED actually fell. Specifically, the ED admission rates decreased from 19.4% to 17.5%, representing a 9.8% relative decline [15]. This decline in admission rates despite an increase in ED visits suggests improvements in ED efficiency, changes in patient case mix, or shifts in hospital admission policies. It also reflects the evolving landscape of emergency care, where hospitals may be adopting new strategies to manage the growing patient load in EDs more effectively, possibly through enhanced triage processes or increased utilization of alternative care settings. Admission rates should mirror the balance between case severity and ED capacity. However, managing a high influx of severe cases is challenging, as it can disrupt patient flow and strain resources. Admission decisions also face variability and potential subjectivity, impacting the accuracy of this measure. Accurate and swift assessments are crucial to determine the necessity for admission while avoiding over or under-admitting patients [16].

Resources: Resources in the ED context incorporate the availability and allocation of medical equipment, medication, staffing, and facilities necessary to deliver timely and effective emergency care. The adequacy and quality of these resources directly influence the ED's ability to respond to patient needs efficiently and effectively. Resource adequacy encompasses not only the quantity but also the appropriateness of medical supplies and personnel skills [7]. The ideal scenario is to have a well-equipped ED with sufficient, well-trained staff and a steady supply of necessary medical equipment and medication. However, there are several challenges, including budget constraints, resource allocation, maintaining up-to-date equipment, and ensuring adequate staffing levels, especially during peak times or crises. Optimal resource management is essential for reducing wait times, preventing overcrowding, and improving overall patient outcomes and satisfaction [17]. Müller et al. focused on predicting the total resource consumption in EDs, they aimed to analyze and forecast ED resource usage based on patient characteristics and consultation details. They found that accurately predicting resource needs can greatly assist in resource planning and allocation, thereby improving the efficiency and quality of care in the ED setting [18]. Etu et al. showed that proper management and deployment of resources are critical in enhancing ED performance, particularly in high-demand situations [19]. According to Aaronson et al., resource management, including staffing and equipment, plays a significant role in determining the quality of care provided in EDs [20].

Process

Time to diagnosis, time to treatment, and time to pain management: Time to diagnosis, time to treatment, and time to pain management are critical process indicators in emergency care. These metrics are measured from the patient's arrival to the moment they receive a diagnosis, start treatment, or receive pain management. Patients are typically assessed promptly upon arrival, with guidelines recommending assessment within 15 minutes of arrival in many cases [21]. Triage nurses prioritize patients based on the severity of their condition. After triage, a healthcare provider conducts an initial evaluation to determine the patient's condition. This step should be done promptly to identify critical cases [21]. Once a patient's condition is assessed, diagnostic tests such as blood tests, imaging, or ECG may be ordered. The time for diagnostic testing varies based on the urgency and complexity of the case. Timely treatment is crucial, especially for critical conditions. The goal is to start treatment as soon as possible, and this can vary from immediate interventions for life-threatening cases to a more extended timeline for non-urgent situations [3]. For certain conditions like chest pain, achieving a door-to-ECG time of 10 minutes is a goal to ensure timely evaluation and treatment [22]. Challenges include managing varying patient acuity levels, ensuring timely and accurate triage, and maintaining efficient workflow in the ED to prevent bottlenecks that delay care. Efforts are made to minimize delays in both arrival and treatment to ensure that patients receive timely care and reduce potential adverse outcomes [3].

ED length of stay/wait time: The length of stay (LOS) or wait time in an ED is a critical indicator of both performance and quality of care. It not only reflects the efficiency of the department in delivering timely care but also directly influences patient satisfaction and clinical outcomes [23]. Extended wait times are often associated with patient dissatisfaction and can negatively impact their overall experience in seeking emergency care. Efficiently managing LOS is vital as it allows for better resource allocation, including optimal utilization of staff, beds, and medical equipment, especially in high-pressure emergency situations [24,25]. Timely assessment and prompt treatment are crucial in improving patient outcomes, as delays can exacerbate medical conditions and increase the risk of complications. Internationally, there are established benchmarks for acceptable ED LOS, with a target for most patients to complete their ED journey within four hours [26,27]. However, the actual LOS can vary significantly among different EDs, influenced by a myriad of factors such as patient volume, staffing levels, and the efficiency of operational processes [19]. Addressing these factors is essential for any ED aiming to enhance its service quality and meet the set international standards.

Correct diagnosis and appropriate treatment: Correct diagnosis and appropriate treatment are critical indicators of ED performance. These indicators help assess the efficiency of emergency care [19]. EDs should efficiently recognize and prioritize patients with time-dependent critical conditions. Rapid assessment and triage are essential to ensure timely treatment [28]. Correct diagnosis is crucial as it sets the foundation for the entire treatment plan, influencing patient outcomes and the efficacy of the treatment administered. Efficient diagnosis contributes to smoother patient flow within the ED, reducing overcrowding and improving the overall patient experience. This can be a key performance indicator (KPI) for EDs seeking to optimize their operations [1]. Appropriate treatment, on the other hand, ensures that patients receive the most suitable medical interventions based on their diagnosis, which is essential for improving recovery rates and reducing the likelihood of complications. Challenges in maintaining high standards in these areas include the wide range of medical conditions presented in EDs, the time-sensitive nature of emergency care, and the need for continual training and updates in medical knowledge and practices [29]. Achieving high rates of correct diagnosis and appropriate treatment is a hallmark of quality in emergency care, significantly impacting patient satisfaction, safety, and overall ED efficiency. Continuous monitoring and analysis of these indicators are essential for ongoing quality improvement in emergency care [2].

Physician workload: Physician workload in the ED is a critical process indicator, impacting both the quality of care provided and the well-being of the medical staff. High physician workloads can lead to burnout, stress, and decreased job satisfaction. This can negatively affect the mental well-being of physicians, potentially compromising their performance and decision-making abilities in critical situations [30]. Overworked physicians may have limited time and attention to devote to each patient, potentially leading to longer wait times, reduced patient satisfaction, and lower quality care [31]. This can impact the overall patient experience in the emergency department. Moreover, Increased workload can contribute to higher rates of medical errors, including misdiagnoses, medication errors, and procedural mistakes [32]. These errors can have serious consequences for patient safety and outcomes. Various studies have explored methods to measure and track physician workload in the emergency department. These measurements can help in understanding workload patterns, optimizing staffing, and improving physician performance [33-36]. Physician workload can also be affected by interruptions during their shifts. Managing interruptions effectively is crucial for maintaining focus and managing patient care efficiently [33]. Workload balancing is a key consideration in emergency department physician rostering. Efficient scheduling and distribution of workload among physicians can help optimize performance and resource utilization [36].

Standard of care treatment: Standard of care treatment is a benchmark that defines the level of care and treatment that is expected to be provided to patients based on current medical knowledge and best practices. Its importance lies in its role as a metric for evaluating the quality of care provided in EDs [37]. High adherence to the standard of care ensures that patients receive treatments that are both effective and aligned with the latest medical guidelines. This adherence is crucial in the ED setting, where decisions and actions must be taken swiftly and accurately to address a wide range of urgent health issues [38]. By measuring the standard of care in EDs, healthcare providers and administrators can assess how well their services meet established medical guidelines, which is critical for patient safety and treatment efficacy [39]. The use of the standard of care as an indicator of ED performance is multifaceted. Firstly, it provides a clear framework for healthcare providers in delivering care, ensuring that all patients receive a uniform level of treatment regardless of their presenting condition or the individual healthcare provider. This consistency is vital in emergency settings where the complexity and variability of cases can be challenging. Secondly, standard-of-care treatment metrics can serve as a tool for continuous improvement. By regularly assessing adherence to these standards, EDs can identify areas where they are excelling and areas that require improvement. This ongoing evaluation is essential for maintaining high-quality care and for adapting to new medical discoveries and treatment methodologies.

Quality of care measure: The use of "quality of care" as an indicator of ED performance has become increasingly vital in evaluating and enhancing healthcare services. Quality of care encompasses a range of factors, including patient outcomes, satisfaction, and adherence to clinical guidelines. It serves as a comprehensive measure, reflecting not only the clinical effectiveness of treatments but also patient experiences and operational efficiency. Studies have shown that focusing on quality of care can lead to significant improvements in ED performance. For example, Schull et al. highlighted the necessity of developing new indicators in various domains like patient satisfaction and mental health to enhance the overall quality of care in EDs. They also mentioned the challenges in evaluating ED quality due to the lack of agreement on appropriate measures, advocating for standardized, evidence-based quality of care indicators [40]. Konson et al. demonstrated minimal changes in compliance rates with quality of care indicators, suggesting that once a high standard of care is achieved, maintaining it becomes crucial [41]. Further studies reinforce the importance of evidence-based indicators in assessing ED quality. A comprehensive systematic review emphasized the need for performance indicators in EDs that are grounded in robust evidence, highlighting how these metrics can lead to more informed and effective healthcare practices [42]. These studies collectively underscore the critical role of quality of care as a multifaceted and dynamic performance indicator, essential for the continuous improvement and benchmarking of ED services.

Outcome

ED returns: The concept of ED return visits, especially within a 72-hour window, has been extensively studied as an indicator of ED performance. Several studies have highlighted a link between the characteristics of the patient population in a given ED and the likelihood of patients returning within a 72-hour window [43,44]. Some of these relationships might not be immediately apparent. For instance, after a pediatric ED transitioned from free-text to structured discharge instructions, there was a notable 30% surge in return visits, but without any discernible impact on patient safety [45]. In a different pediatric investigation, the frequency of physician-specific 72-hour returns was found to be the least related to clinical performance out of seven evaluated markers [46]. Two studies have concurred that the frequency of ED return visits does not necessarily indicate a shortfall in care quality. An extensive retrospective study employing data from the National Hospital Ambulatory Medical Care Survey revealed that patients who came back for a second ED visit within 72 hours were neither more severely ill nor more likely to require hospital admission than other ED patients [47]. Another study, conducted across three hospitals in 2013, discovered that 95% of patients returning within 72 hours and subsequently admitted had no identifiable care deficiencies during their initial visit. This research group posited that data on 72-hour returns would not effectively measure the overall quality of care unless accompanied by a detailed, manual review of patient records [47]. The study by Shy et al. delves into this concept, showing that such return visits are often used as a screening tool in quality assurance processes to identify high-risk cases and potential lapses in care, rather than as a direct measure of ED performance [48]. They emphasize the need for a more nuanced approach to evaluating 72-hour return cases. They suggest a bi-level review process, involving both residents and faculty in the quality assurance process, to review all cases of return visits. This approach aims to balance thoroughness with feasibility, given the resource constraints in many ED settings. The study also highlights the need for additional research to establish best practices for conducting these reviews. Overall, while 72-hour returns can be a valuable tool in identifying areas for improvement in ED care, their use as a standalone metric for ED performance is limited. The study calls for a more comprehensive approach to quality assurance in emergency medicine, one that considers a wider range of indicators beyond just return visit frequency [48].

Left without being seen (LWBS): The metric of patients' LWBS in EDs is increasingly recognized as a critical indicator of ED performance. This measure is intricately linked to multiple operational aspects within the ED, including the efficiency of patient flow, the length of waiting times, and the overarching patient satisfaction levels. A higher incidence of LWBS often signals potential issues in the quality and safety of care provided in the ED. For instance, a study by Li et al. highlighted this connection, indicating that a high LWBS rate could be symptomatic of deeper operational challenges, particularly in ensuring timely care for all patients [49]. Similarly, another study showed that LWBS is associated with higher ED return rates and admission rates, resulting in a financial impact on the healthcare systems [50]. Moreover, LWBS rates are frequently correlated with the issue of ED overcrowding. Overcrowding not only affects the quality of patient care but also the working conditions for staff, leading to a cascade of operational inefficiencies. This relationship was emphasized in a study conducted in Saudi Arabia, which used the LWBS rate as a key performance marker, underscoring how overcrowding can lead to increased LWBS incidents [51]. Such a trend makes LWBS an invaluable metric for assessing how well an ED is managing its patient load and the effectiveness of its operational strategies.

Mortality: The use of mortality rates as an indicator of ED performance has become a significant point of focus in healthcare quality and performance assessment. Mortality rates, especially in the context of EDs, provide critical insights into the effectiveness of emergency care, patient outcomes, and overall hospital performance. For instance, the Mortality in Emergency Department Sepsis (MEDS) score, as studied by Jones et al., is specifically designed to predict mortality among ED patients with sepsis, highlighting the importance of specific diagnostic tools in evaluating ED performance [52]. This demonstrates how mortality rates can be used to assess the quality of care and the accuracy of diagnoses in emergency settings. Furthermore, the relationship between ED stay duration and mortality rates is another aspect that is frequently studied. Wessman et al. found that primary ED diagnoses were a reliable predictor of short-term mortality [53]. This relationship underscores the importance of efficient and accurate diagnosis and treatment in EDs, as prolonged stays or misdiagnoses can adversely affect patient outcomes. Moreover, mortality rates are also used to evaluate the impact of systemic factors on ED performance. For instance, Richardson et al. linked increased patient mortality with ED overcrowding, indicating how systemic issues like overcrowding can directly impact patient outcomes [54]. Lastly, mortality rates in EDs serve as a benchmark for hospital-wide quality improvement initiatives. Berthelot et al. suggested using the Emergency Department Hospital Standardized Mortality Ratio (ED-HSMR) to measure the performance of hospital systems in emergency care and guide quality improvement efforts [55]. This kind of measure provides a quantitative basis for assessing the effectiveness of care and identifying areas needing improvement. Overall, mortality as an indicator offers a multifaceted view of ED performance, encompassing clinical, operational, and systemic aspects of healthcare delivery.

Satisfaction

Patient satisfaction: Patient satisfaction in the ED is a crucial metric for evaluating the quality of care and overall efficiency of healthcare services. This measure reflects various aspects of the patient experience, from the quality of medical care received to communication and interaction with healthcare providers. A study by Abass et al. showed that certain areas, such as satisfaction with pain management and medication information, had relatively lower scores, indicating areas for potential improvement [56]. Patient satisfaction is also closely linked to patient compliance, as patients who are more satisfied with their care are more likely to follow medical advice and treatment plans, as highlighted in resources on improving patient satisfaction in the ED [57]. Furthermore, patient satisfaction can influence various other aspects of ED operations. Taylor et al. noted that increased patient satisfaction could lead to improved staff morale and job satisfaction and reduce the likelihood of patients seeking further opinions outside the ED [58]. Moreover, patient satisfaction surveys and their methodologies, as discussed in an information paper, play a significant role in shaping the strategies for enhancing ED performance [59]. These surveys provide valuable feedback on patient experiences and perceptions, guiding EDs in identifying areas for improvement and implementing necessary changes.

Peer-assessed physician performance: Peer-assessed physician performance is an increasingly utilized method for evaluating and enhancing the quality of medical care, particularly in EDs. This form of evaluation involves medical peers and colleagues providing feedback on a physician's clinical and non-clinical performance. Etherington et al. demonstrated this approach by having emergency physicians in an urban ED evaluate their peers through a survey covering various aspects of effectiveness [60]. Similarly, Alameddine et al. discussed the use of peer evaluation to assess physicians’ interpersonal and communication skills, underscoring its significance in comprehensively evaluating a physician's capabilities beyond just clinical expertise [61]. Moreover, multisource feedback (MSF), as described in a study, is another form of peer assessment where surveys are administered amongst medical peers and colleagues to evaluate physicians [62,63]. This comprehensive feedback mechanism provides a holistic view of a physician's performance, including their clinical skills, communication, teamwork, and professional behavior [64]. Such peer assessments are crucial for continuous professional development, helping to identify areas of strength and opportunities for improvement in a physician's practice [65].

Provider communication: Provider communication in the ED is a fundamental aspect of patient care and significantly impacts patient satisfaction and overall ED performance. Effective communication between healthcare providers and patients in the ED setting is challenging yet essential for ensuring quality care. A study by Degabriel et al. emphasized the importance of the doctor-patient relationship in the ED, highlighting how communication can be challenging in this high-pressure environment [66]. Gabay et al. further underscored that communication is a key element of patient satisfaction in the ED, with positive interpersonal exchanges playing a significant role in the overall experience of patients [67]. Moreover, the complexity of communication in the ED, involving multiple interactions between nurses, physicians, and other clinicians, is a critical factor for efficient ED operations [68]. Patterson et al. highlighted the intricate nature of these communications, stressing the importance of a clear understanding of communication dynamics for effective teamwork in the ED [69]. Scheder-Bieschin et al. pointed out the frequent challenges in communication between patients and healthcare professionals in the crowded ED setting, emphasizing the need for improvement strategies to enhance patient-doctor communication [70].

Rate of complaints: The rate of complaints in the ED is a critical indicator of service quality and patient satisfaction. This metric offers valuable insights into areas needing improvement within ED operations. Griffey et al. emphasized that patient complaints are well-established indicators of quality, highlighting their importance in assessing the performance of emergency care [71]. The analysis of complaints provides a direct reflection of patient perceptions and experiences, offering a unique perspective on the effectiveness of care delivery. Schwartz et al. used statistical methods to associate certain characteristics with a higher risk of complaints, demonstrating the potential of complaints data in identifying specific issues within the ED environment [72]. Such analyses can pinpoint areas like organizational logistics or patient-provider interactions where enhancements are needed. In the context of healthcare KPIs, the percentage of patient complaints relative to total patient visits is a critical measure [73]. It not only assesses patient dissatisfaction but also gauges the overall quality of medical services, as outlined in performance management literature [74,75].

Provider satisfaction: Provider satisfaction in healthcare, particularly in EDs, is a crucial factor that significantly impacts the quality of care and overall operational efficiency. Provider satisfaction encompasses the well-being and contentment of healthcare professionals, including doctors, nurses, and support staff, in their work environment. High provider satisfaction is often linked to improved patient care, as satisfied healthcare providers are more likely to be engaged, motivated, and attentive to patient needs. Moreover, provider satisfaction is an important metric for assessing the work environment and organizational health of the ED, as it directly influences staff turnover rates, job performance, and the propensity for medical errors. While the search results provided do not directly address studies on provider satisfaction, the implications of such satisfaction are clear. Satisfied providers typically contribute to a positive patient experience, as seen in the context of patient satisfaction studies [76]. Furthermore, provider satisfaction is intricately linked to the efficiency of ED operations, as satisfied staff are more likely to contribute positively to patient flow and quality of care [68]. Ensuring high levels of provider satisfaction is crucial for maintaining a high standard of care in the ED, which is a fast-paced and often high-stress environment.

Staff safety: Staff safety in EDs is a critical aspect of healthcare that impacts not only the well-being of healthcare providers but also the quality of patient care [77]. The complex and often high-pressure environment of the ED can pose various safety challenges for staff, including physical and psychological risks. Psychological safety, particularly, is a significant concern, as highlighted by Purdy et al. [78]. Their study found that psychological safety was not uniformly experienced by ED staff, especially among nurses and new employees, indicating a need for better support systems and work environment improvements [78]. Additionally, the overall safety of the ED environment is crucial for staff performance and morale. Factors such as inadequate training, staff burnout, low morale, and insufficient remuneration can significantly affect staff safety and, by extension, the quality of care provided in the ED [79]. Addressing these issues is essential for maintaining a safe and effective work environment. Strategies to improve ED performance often include interventions focusing on practice changes and process enhancements, which can also contribute to enhancing staff safety [68]. Ensuring staff safety in the ED is not just about preventing physical harm; it also involves creating a supportive environment where staff feel secure and valued. This holistic approach to staff safety is crucial for fostering a high-performing and patient-centered ED.

Strategies to improve ED performance

Strategies to Improve ED Structures

Reducing ED occupancy and crowding is crucial for enhancing overall ED performance and patient outcomes. One effective strategy is the implementation of a fast-track system for patients with non-critical conditions [80]. This approach involves triaging patients upon arrival to identify those with less severe issues who can be seen quickly by a dedicated team of healthcare providers. This system not only expedites care for those with minor ailments but also frees up resources for more critically ill patients [81]. Additionally, integrating advanced triage protocols can significantly reduce wait times and improve patient flow [82]. By employing skilled nurses or practitioners to conduct initial assessments and initiate necessary tests or treatments at the triage stage, hospitals can streamline the process, leading to quicker diagnosis and treatment [83,84]. Another key strategy involves enhancing coordination and communication between the ED and other hospital departments. Optimizing bed management and ensuring timely patient transfers can substantially reduce ED crowding. This can be achieved through the use of real-time bed tracking systems and implementing efficient discharge processes in inpatient units to make beds available more quickly [85]. Furthermore, expanding the role of multidisciplinary teams, including case managers and social workers, can address non-medical issues that often contribute to prolonged ED stays, such as arranging follow-up care or addressing social determinants of health. By focusing on these areas, hospitals can not only improve ED performance but also enhance the overall patient experience and outcomes.

To enhance admission rates in ED, it's essential to focus on strategies that streamline patient flow and improve the accuracy of diagnosis and treatment [1]. One pivotal approach is the implementation of advanced diagnostic protocols and tools. Utilizing state-of-the-art diagnostic equipment and adopting evidence-based clinical decision rules can aid in the rapid and accurate identification of conditions that require admission. Moreover, training ED staff in the latest diagnostic techniques ensures that patients are efficiently evaluated, leading to quicker decision-making regarding admissions [86]. Another crucial aspect is the integration of EHRs and telemedicine. EHRs enable better tracking of patient history and symptoms, facilitating more informed decisions about admissions. Telemedicine, on the other hand, can be used for remote consultations with specialists, ensuring that expert opinions are readily available, which can expedite the admission process for patients needing specialized care [87]. Another key strategy involves enhancing communication and collaboration with inpatient units and other hospital departments. Efficient communication channels can significantly reduce the time taken to transfer patients from the ED to appropriate inpatient units, thereby improving bed availability and overall admission rates [85]. Furthermore, regular training and workshops for ED staff on patient assessment and inter-departmental coordination can lead to a more cohesive approach to patient care, thus optimizing the admission process. By focusing on these strategies, emergency departments can not only enhance their admission rates but also improve the quality of care and patient outcomes.

Optimizing resources in the ED is vital for improving efficiency and patient care quality [88]. A primary strategy involves the effective management of human resources. This includes implementing flexible staffing models that align with patient inflow patterns. By analyzing historical data, EDs can predict peak times and ensure adequate staffing during these periods, reducing wait times and enhancing patient throughput [89]. Additionally, cross-training staff to perform multiple roles can increase flexibility and efficiency, allowing the ED to adapt quickly to changing patient needs [90]. Another aspect of resource optimization is the utilization of lean management principles. This involves identifying and eliminating inefficiencies in patient flow and processes, such as redundant paperwork, unnecessary steps in patient care, and bottlenecks in patient movement. Implementing process improvements like bedside registration and point-of-care testing can significantly reduce turnaround times and enhance patient care efficiency [91]. Technological advancements also play a crucial role in resource optimization. Investing in advanced medical equipment and healthcare IT systems, such as EHRs and real-time tracking systems, can streamline operations and improve data access and accuracy. EHRs enable quick access to patient information, reducing duplication of tests and procedures, while tracking systems can help manage bed availability and patient flow more effectively. Moreover, embracing telemedicine can optimize resource use by allowing remote consultations, reducing the need for specialist physical presence for certain diagnoses or follow-ups [92]. Lastly, fostering a culture of continuous improvement and innovation within the ED staff can lead to the regular identification and implementation of new strategies for resource optimization. By focusing on these approaches, EDs can significantly enhance their operational efficiency, resource utilization, and ultimately, patient care quality, as shown in Table 1.

Strategies to Improve the ED Process

To minimize the time to diagnosis, the implementation of rapid assessment and triage protocols is essential. This involves training staff to quickly identify the severity of a patient's condition upon arrival and prioritize accordingly. Utilizing point-of-care testing (POCT) and advanced diagnostic tools can also expedite the diagnostic process. These technologies enable healthcare providers to perform critical tests and obtain results directly at the bedside, significantly reducing the waiting time for lab results [93]. Furthermore, integrating decision support systems within EHRs can assist physicians in making quicker and more accurate diagnoses based on patient data and evidence-based guidelines [94]. Reducing time to treatment involves streamlining the process from diagnosis to the initiation of appropriate medical interventions. This can be achieved by enhancing communication and coordination among ED staff, ensuring that once a diagnosis is made, treatment plans are promptly developed and executed [95,96]. Adopting standardized treatment protocols for common conditions can also expedite care delivery, as they provide clear guidelines for immediate action. Additionally, ensuring the availability of essential medications and equipment within the ED avoids delays caused by having to source these from other parts of the hospital [39]. Effective pain management is a critical component of patient care in the ED. To reduce the time to pain management, EDs can implement protocols for early pain assessment and treatment. This includes training staff to promptly identify and assess pain levels as part of the initial patient evaluation [97]. Establishing guidelines for the use of analgesics and non-pharmacological pain relief methods ensures that patients receive timely and appropriate pain management. Furthermore, involving patients in pain management decisions and educating them about the options available can lead to more effective and personalized care [98]. Reducing the length of stay and wait time in the ED is a critical factor in improving patient satisfaction and throughput. Key to this is the implementation of efficient triage systems that prioritize patients based on the severity of their conditions, ensuring that those in need of urgent care are attended to promptly. Streamlining processes through the integration of advanced technology, such as EHRs and real-time tracking systems, can significantly reduce administrative delays and enhance communication among staff. Additionally, adopting lean management principles to identify and eliminate bottlenecks in patient flow, and ensuring adequate staffing based on predictive patient inflow models, are essential.

Effectively managing physician workload in the ED involves a multi-faceted approach. Predictive analytics aid in anticipating patient inflow, facilitating optimal resource deployment [99]. Delegating routine tasks to support staff, like medical scribes, eases physicians' non-clinical burden [100]. Implementing wellness programs addresses physician burnout, promoting a healthier work-life balance. Soliciting regular feedback fosters continuous improvements in workload management [101,102]. Additionally, optimizing patient flow through efficient capacity management, such as bed availability and discharge processes, alleviates pressure on physicians, contributing to improved patient care and departmental efficiency.

Strategies to Improve ED Outcomes

Improving ED returns involves strategic measures to ensure effective follow-up and continuity of care. First, implementing robust discharge planning and education is crucial. This includes providing patients with clear instructions about their diagnosis, treatment plan, and when to seek further medical attention [103]. Enhancing communication channels for follow-up care, such as scheduled calls or telehealth appointments, can also ensure patients understand and adhere to their treatment plans, reducing the likelihood of return visits. Additionally, integrating a comprehensive EHR system helps in tracking patient outcomes and identifying those at higher risk of return, facilitating targeted interventions [104].

Addressing the issue of patients LWBS requires a focus on reducing wait times and streamlining patient flow. Initiating a rapid triage system ensures that patients are assessed and categorized based on the urgency of their condition upon arrival. Implementing fast-track lanes for less severe cases can significantly decrease wait times for these patients, while simultaneously freeing up resources for more critical cases. Enhancing staff efficiency through additional training and optimizing staffing levels, especially during peak hours, can further reduce bottlenecks and improve overall patient throughput [105,106].

To improve mortality rates in the ED, a multifaceted approach is needed. This includes the adoption of advanced resuscitation techniques and ensuring that staff are trained in the latest life-saving protocols. Quick access to critical care resources, such as life-support equipment and medications, is essential. Furthermore, fostering a culture of continuous learning and improvement is vital, which can be achieved through regular training sessions and mortality reviews to learn from past cases. Enhancing coordination with other departments like intensive care units for the timely transfer of critically ill patients can also play a crucial role in improving survival rates. These strategies, combined with a focus on patient-centered care and the use of evidence-based practices, can contribute significantly to reducing mortality rates in the ED [107,108].

Strategies to Improve ED’s Patient and Physician Satisfaction

Improving patient and physician satisfaction in the ED requires a holistic approach focused on efficiency, communication, and work environment. For patient satisfaction, key strategies include reducing wait times and enhancing the quality of care. Implementing efficient triage systems and fast-track lanes for less critical cases can significantly decrease waiting periods. Improving the communication skills of the medical staff ensures that patients feel heard and understood, which is crucial for their satisfaction. Providing clear, concise information about their condition, treatment, and expected wait times helps in setting realistic expectations. Additionally, creating a comfortable waiting area with amenities like Wi-Fi and updated information displays can improve the overall patient experience.

Physician satisfaction can be enhanced by addressing workload management and providing a supportive work environment. Optimizing staffing levels to match patient influx patterns helps prevent burnout. Offering professional development opportunities, including training in the latest medical advancements and leadership skills, fosters a sense of growth and accomplishment [109]. Implementing efficient administrative processes and utilizing technology like EHRs and decision support tools can reduce paperwork and allow physicians more time for patient care. Creating a culture of feedback and open communication where physicians feel their input is valued and acted upon is also crucial [110]. Moreover, fostering a collaborative team environment where all staff members, including nurses, technicians, and support staff, work together seamlessly can significantly impact overall satisfaction. Recognizing and celebrating successes and providing support during challenging times contribute to a positive work culture. Implementing these strategies not only improves the satisfaction levels of both patients and physicians but also enhances the overall efficiency and effectiveness of the ED.

## Conclusions

In conclusion, this study has systematically outlined a comprehensive range of strategies to enhance various aspects of ED performance, spanning from structural improvements to process optimizations, outcome enhancements, and satisfaction measures. For the ED structure, strategies like fast-track systems for minor ailments, advanced diagnostic tools, and resource optimization through flexible staffing models and lean management principles were emphasized. In terms of ED processes, the focus was on reducing the time to diagnosis and treatment, streamlining patient flow to decrease wait times, and ensuring correct diagnoses and appropriate treatments through continuous medical education and evidence-based protocols. The study also addressed critical outcomes like reducing ED return visits, managing patients who have LWBS, and improving mortality rates through advanced resuscitation techniques and better coordination with intensive care units. For enhancing ED satisfaction, strategies were centered on improving both patient and provider experiences. This included measures to reduce wait times, enhance communication skills, offer professional development opportunities for staff, and create a supportive work environment. Collectively, these strategies offer a multifaceted approach to improving ED performance. By implementing these measures, EDs can not only enhance their operational efficiency but also significantly improve the quality of patient care and satisfaction, ultimately leading to better health outcomes and a more sustainable healthcare system.
